# Folic Acid Affects Iron Status in Female Rats with Deficiency of These Micronutrients

**DOI:** 10.1007/s12011-019-01888-z

**Published:** 2019-09-11

**Authors:** Joanna Suliburska, Katarzyna Skrypnik, Agata Chmurzyńska

**Affiliations:** Institute of Human Nutrition and Dietetics, Poznan University of Life Sciences, Wojska Polskiego St. 31, 60-624 Poznan, Poland

**Keywords:** Folic acid, Iron, Supplementation, Rats, Iron deficit

## Abstract

Although simultaneous supplementation with iron and folic acid is justified, the potential interactions between these micronutrients are unknown. The aim of this study was to determine the effects of oral iron and folic acid, administered together or separately, on iron concentration in tissues in rats with a deficiency of both these micronutrients. In the first stage of the experiment (28 days), 150 8-week-old female Wistar rats were randomly assigned to a control group (C; *n* = 30) fed the standard diet and to a study group (*n* = 120) fed a diet deficit in iron and folate. The study group was then randomly divided to four groups: D group fed a deficit diet, FE group fed a deficit diet with iron gluconate, the FOL group fed a deficit diet with folate acid, and the FEFOL group fed a deficit diet with iron gluconate and folate acid. After 2, 10, and 21 days of supplementation, ten animals from each group were killed. Morphological parameters were measured in whole blood. Iron concentration was assayed in serum, liver, spleen, pancreas, heart, and kidneys. Folic acid supplementation more significantly decreased iron concentrations in the pancreas and spleen than in the D group after 10 and 21 days of supplementation. Moreover, the combination of iron with folic acid markedly decreased iron levels in the liver and spleen, in comparison with iron alone, after 10 and 21 days of the experiment. In conclusion, folic acid affects iron status in female rats deficient in these micronutrients in moderate and long-term supplementation.

## Introduction

It has been shown that iron and folate are of great importance for women of reproductive age. The availability of sufficient quantities of these micronutrients plays a critical role in pregnancy and fetal development. Inadequate intake levels of these micronutrients among young women has been documented in many developed countries [1, 2].

Low levels of iron and folate have been also found in the daily food rations of pregnant women, which is associated with an increased risk of anemia for the mother and of neural tube defects for the fetus [2–4]. The World Health Organization (WHO) has estimated that iron deficiency anemia (IDA) affects approximately 146.5 million young children and 234 million nonpregnant women in the world, and constitutes an enormous public health problem leading to high mortality, especially in developing countries [3, 4]. The most detrimental effects of IDA are immunological and neurological disorders, impairment of thermoregulation, cognitive dysfunction, fatigue, and inhibition of psychomotor development [5]. Women are generally more vulnerable to Fe deficiency than men [5, 6]. Due to its harmful health effect, Fe deficiency undoubtedly requires effective treatment. Folate deficiency leads to impaired DNA synthesis and increased death of hematopoietic cells, eventually resulting in anemia due to ineffective erythropoiesis. Large quantities of iron are also required for hemoglobin synthesis [7]. Since folate and iron are required for effective erythropoiesis, the World Health Organization (WHO) has recommended daily oral iron and folic acid supplementation as part of antenatal care, in order to reduce the risks of low birth weight, maternal anemia, and iron deficiency [3, 4].

Experts in gynecology and nutrition have emphasized the importance of FA and Fe supplementation among women of reproductive age. However, increasing the intake of nutrients or using supplements is not always effective [8, 9]; its effectiveness depends on factors that affect the bioavailability of minerals and vitamins, which include the food matrix (i.e., interactions with other nutrients that may increase or decrease the absorption of folate or Fe, such as vitamin C and phytates [10]) and the functioning of body, which in turn depends partly on genotype and gut microbiota [11]. Thus, the effects of adding folic acid to oral Fe supplementation, as well as its possible favorable or disadvantageous consequences, especially under conditions of Fe deficiency, remain unknown.

The aim of this study was to investigate the effect of oral Fe and folic acid administered together or separately on the liver, kidney, heart, spleen, and pancreas Fe content in rats with deficiencies of these micronutrients.

## Material and Methods

### Animals

A total of 150 8-week-old female Wistar rats purchased from AnimaLab (Germany) were used. The mean weight of the rats was 182.9 g. The animals were housed in stainless steel cages coated with metal-free enamel and kept under cycles of 12 h light and 12 h dark. Room temperature was maintained at 20 ± 1 °C with 5565% humidity. The animal procedures were approved by the local bioethics committee (approval no. 59/2016).

### Experimental Design

The animals were adapted to laboratory conditions during the first 5 days. In the first stage of the experiment, the animals were randomly assigned to two groups, the first of which contained 120 rats and the second of which had 30 rats. The animals were fed a semisynthetic diet based on the AIN-93M diet [12]. Rats in first group were fed an iron and folate deficit diet, while those in the second group were fed the standard diet (Fe contents: 56 mg/kg and folic acid contents: 2 mg/kg). In the deficit diet, no iron and folic acid were added to the mineral and vitamin mixture of the AIN-93M diet. All rats were provided ad libitum diet and distilled water for 28 days. After that time, in the second stage, the first group was randomly divided into four groups of 30 rats each. Group D continued to intake the deficit diet, group FE was fed a deficit diet with iron gluconate (150 mg Fe/kg diet), group FOL was fed a deficit diet with folate acid (6 mg/kg diet), and group FEFOL was fed a deficit diet with iron gluconate (150 mg Fe/kg diet) and folate acid (6 mg/kg diet). Group C was fed the standard diet. The intake of the diets was monitored daily.

After 2 days, and then after 10 days and 21 days of the experiment, ten animals of each group were anesthetized and killed by cardiac puncture (after 12 h of fasting).

The liver, kidney, heart, spleen, and pancreas were dissected, weighted, and stored at − 80 °C. The initial and final body weights were measured using electronic scale.

### Determination of Iron and Morphological Parameters

Iron concentration in serum was determined using a commercial kit (Thermo Scientific, Vantaa, Finland). The iron content of the diet and tissues was determined following digestion in 65%(w/w) spectra pure HNO_3_ (Merck) in the Microwave Digestion System (Speedwave XPERT microwave digestion system, Berghof, Eningen, Germany). Thereafter, the concentration of iron was measured using flame atomic absorption spectrometry (Atomic Absorption Spectrophotometer ZA3000, Hitachi, Tokyo, Japan). The accuracy of the method was verified using certified reference materials: Brown Bread BCR191 for the diet samples, Sigma-Aldrich and bovine liver-trace elements, NIST-1577C, CERT, for tissue samples. The accuracies proved to be 94% and 95.5%, respectively.

Whole-blood morphological analysis (hemoglobin (HB), hematocrit (HCT), red blood cells (RBC), and mean corpuscular volume (MCV)) was performed by a commercial laboratory with the use of SYSMEX XT-4000 (Synevo, Poznań, Poland).

### Statistical Analysis

Detailed statistical analysis was performed using Statistica for Windows12.0. (StatSoft, Poland). The results were expressed as arithmetic means with standard errors. One-way analysis of variance (ANOVA), followed by a post-hoc Turkey’s test, were used to compare the data between groups. Student’s *t* test was used to compare the two selected groups (D and FOL, FE and FEFOL). A Pearson correlation test was carried out to calculate correlations coefficients. The significance was set to the *p* < 0.05 level.

## Results

**Fig. 1:**
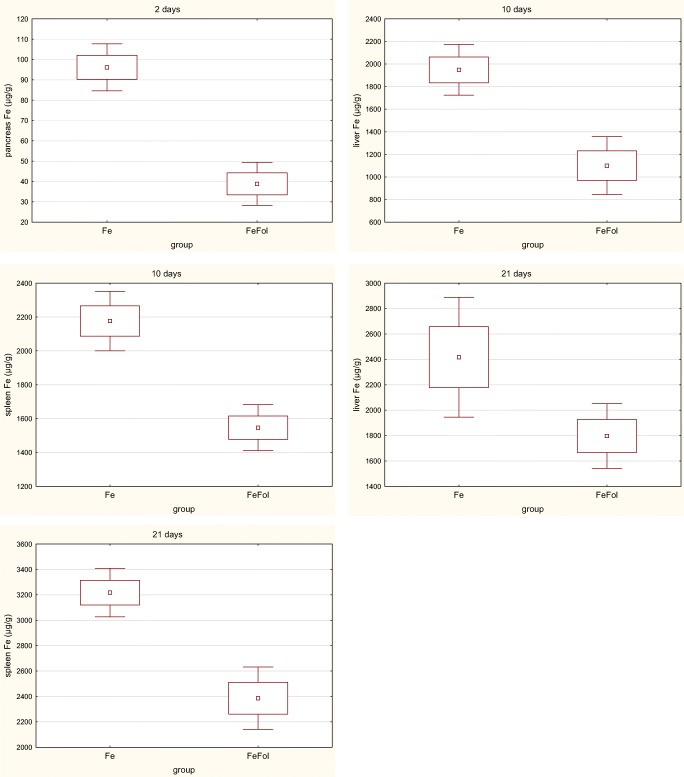
Significant differences between concentration of iron (mean and standard deviation) in tissues in FE and FEFOL groups

**Fig. 2:**
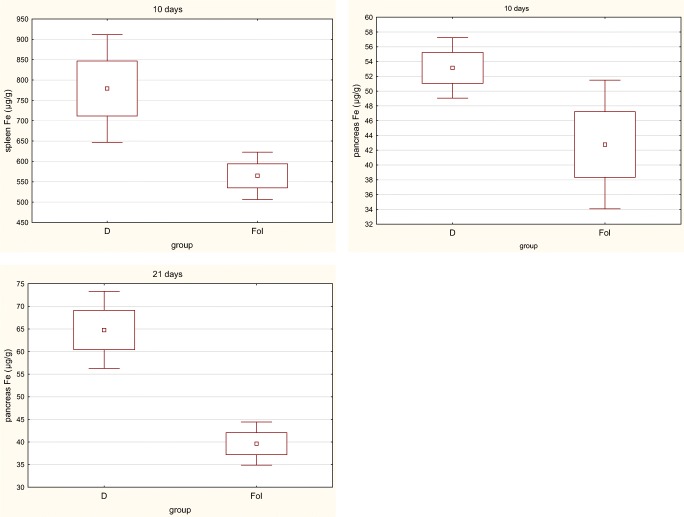
Significant difference between iron concentration (mean and standard deviation) in tissues in D and FOL groups

The results of the study are shown in Tables 1, 2, 3, 4, 5, and 6 and Figs. 1 and 2. The iron concentration measured in the diet samples are shown in Table 1. The daily intake of diet was comparable between groups and the daily intake of iron was around three times higher in the supplemented group than in the control group (Table 2), as assumed. Initial and final body weight did not differ between groups in any period of the experiment. A significant change in relative tissue weight was observed only on day 2 of the experiment. The relative weight of the liver in the FE group was markedly higher than in the FOL group, while the relative weight of the pancreas in the FE group was lower than in the C and FEFOL groups (Table 3). The concentration of iron in serum was comparable between the groups throughout the experiment (Table 4). Significant changes in hemoglobin concentration and hematocrit were observed after 21 days of supplementation with higher level in C and FEFOL groups and lower level in D and FOL groups (Table 5). Markedly differences in MCV values were found in all stages of the experiment and the highest level was observed in C group and the lowest in FOL group. Interestingly, significant correlation between RBC and MCV (*r* = − 0.34) was found only on 21 days of supplementation. This correlation may explain significant changes in HCT with comparable amount of RBC between groups, indicating that a larger number of red blood cells was associated with their smaller volume. On day 2 of the intervention, we observed that the iron concentration in the liver leveled out in the supplemented groups FE and FEFOL (Table 6). In the spleen, kidneys, and heart, the iron level remained lower than in the control group. Short-time supplementation led to the highest level of iron in the pancreas of the FE group and the lowest in the FEFOL group. By day 10 of supplementation, the highest level of iron in the liver was found in the FE group. In the spleen and kidneys, significantly higher levels of iron in were seen in the C, FE, and FEFOL groups than in the D and FOL groups. However, the spleen concentration of iron was markedly higher in the FE and C groups than in the FEFOL group. In the pancreas and heart, the level of iron was higher in the C group than in the other groups (Table 5). For long-term supplementation, we observed significantly higher concentrations of iron in the liver, spleen, and kidneys of the control and supplemented groups than in groups D and FOL. However, liver and spleen iron levels were much higher in the FE group than in the C and FEFOL groups. The lowest pancreatic concentration of iron was seen in the FOL group, while the highest was seen in the C group.

**Table 1:** Content of iron (mean and standard deviation) in the diets

Group	Fe mg/kg diet
C	56.02 ± 0.13
D	6.91 ± 0.24
FE	154.94 ± 1.59
FOL	6.89 ± 0.09
FEFOL	155.42 ± 4.52

**Table 2:** Daily intake of the diet and iron (mean and standard deviation) in rats

Group	Diet (g)	Fe (mg)
	2 days	
C	19.72 ± 0.97	1.10 ± 0.05
D	20.30 ± 0.99	0.14 ± 0.00
FE	19.03 ± 0.60	2.95 ± 0.09
FOL	19.59 ± 0.69	0.13 ± 0.00
FEFOL	20.53 ± 0.72	3.19 ± 0.11
	10 days	
C	20.31 ± 0.86	1.14 ± 0.05
D	20.75 ± 0.81	0.14 ± 0.00
FE	19.54 ± 0.83	3.03 ± 0.13
FOL	19.92 ± 0.33	0.13 ± 0.00
FEFOL	20.28 ± 0.76	3.15 ± 0.12
	21 days	
C	19.91 ± 0.90	1.11 ± 0.05
D	20.18 ± 0.73	0.14 ± 0.00
FE	19.73 ± 0.61	3.06 ± 0.09
FOL	18.96 ± 1.01	0.13 ± 0.00
FEFOL	20.98 ± 0.76	3.16 ± 0.12

**Table 3:** Body weight (g) and relative weight of tissues (% body mass) (mean and standard deviation)

Group	Initial body weight (g)	Final body weight (g)	Liver (%)	Spleen (%)	Pancreas (%)	Kidneys (%)	Heart (%)
	2 days						
C	182.1 ± 10.8	260.9 ± 15.6	2.67 ± 0.19^ab^	0.25 ± 0.04	0.38 ± 0.08^b^	0.68 ± 0.05	0.36 ± 0.02
D	182.3 ± 10.3	267.0 ± 17.2	2.69 ± 0.21^ab^	0.23 ± 0.02	0.38 ± 0.05^ab^	0.67 ± 0.05	0.34 ± 0.04
FE	182.1 ± 10.2	259.3 ± 17.4	2.74 ± 0.39^b^	0.25 ± 0.05	0.30 ± 0.06^a^	0.67 ± 0.04	0.35 ± 0.03
FOL	182.2 ± 10.1	262.7 ± 19.1	2.45 ± 0.25^a^	0.22 ± 0.03	0.35 ± 0.04^ab^	0.66 ± 0.04	0.34 ± 0.03
FEFOL	182.0 ± 10.3	264.7 ± 15.2	2.60 ± 0.31^ab^	0.25 ± 0.02	0.39 ± 0.07^b^	0.69 ± 0.04	0.34 ± 0.02
	10 days						
C	183.0 ± 10.3	274.7 ± 14.7	2.50 ± 0.24	0.23 ± 0.03	0.30 ± 0.07	0.64 ± 0.04	0.33 ± 0.04
D	182.8 ± 10.6	278.9 ± 17.9	2.49 ± 0.20	0.22 ± 0.04	0.35 ± 0.05	0.63 ± 0.12	0.33 ± 0.02
FE	183.0 ± 10.6	267.5 ± 17.2	2.36 ± 0.13	0.21 ± 0.04	0.35 ± 0.04	0.63 ± 0.02	0.32 ± 0.02
FOL	183.0 ± 10.3	275.3 ± 10.7	2.44 ± 0.44	0.24 ± 0.03	0.35 ± 0.07	0.65 ± 0.05	0.33 ± 0.03
FEFOL	183.1 ± 10.4	283.8 ± 22.1	2.34 ± 0.43	0.24 ± 0.03	0.36 ± 0.06	0.65 ± 0.05	0.34 ± 0.03
	21 days						
C	184.0 ± 13.5	289.8 ± 23.5	2.55 ± 0.21	0.25 ± 0.02	0.38 ± 0.13	0.65 ± 0.04	0.35 ± 0.03
D	184.0 ± 13.8	291.4 ± 19.5	2.51 ± 0.28	0.24 ± 0.03	0.34 ± 0.05	0.67 ± 0.05	0.37 ± 0.02
FE	183.7 ± 12.4	288.8 ± 24.8	2.45 ± 0.19	0.22 ± 0.02	0.33 ± 0.04	0.65 ± 0.06	0.35 ± 0.03
FOL	183.6 ± 11.7	286.1 ± 25.8	2.36 ± 0.48	0.21 ± 0.04	0.34 ± 0.04	0.63 ± 0.05	0.35 ± 0.02
FEFOL	183.8 ± 11.8	285.7 ± 22.5	2.38 ± 0.13	0.25 ± 0.03	0.33 ± 0.06	0.60 ± 0.10	0.31 ± 0.08

^a,b,c^Significant differences between groups; ANOVA and Tukey’s test

**Table 4:** Concentration of iron (mean and standard deviation) in serum (μg/ml) in rats

Stage	C	D	FE	FOL	FEFOL
2 days	2.83 ± 0.73	2.41 ± 1.18	3.21 ± 0.72	2.24 ± 0.81	2.74 ± 0.88
10 days	2.40 ± 0.50	2.13 ± 0.75	2.50 ± 1.05	2.19 ± 0.69	2.20 ± 0.53
21 days	2.61 ± 0.84	2.19 ± 0.77	2.13 ± 0.66	1.65 ± 0.59	2.99 ± 0.65

**Table 5:** Concentration of morphological parameters in rats (mean and standard deviation)

Group	HB (g/l)	HCT (l/l)	RBC (× 10^12^/l)	MCV (fl)
	2 days			
C	152.00 ± 3.61	0.47 ± 0.03	8.00 ± 0.24	61.88 ± 1.41^b^
D	146.25 ± 3.30	0.47 ± 0.02	7.80 ± 0.53	59.23 ± 1.12^a^
FE	141.75 ± 4.57	0.46 ± 0.01	7.73 ± 0.31	60.22 ± 1.56^ab^
FOL	147.50 ± 7.78	0.48 ± 0.03	8.05 ± 0.23	58.75 ± 1.38^a^
FEFOL	148.50 ± 4.51	0.48 ± 0.01	8.04 ± 0.36	59.48 ± 1.74^a^
	10 days			
C	151.00 ± 3.46	0.46 ± 0.02	7.97 ± 0.27	61.92 ± 1.42^b^
D	146.25 ± 3.30	0.47 ± 0.01	7.80 ± 0.53	59.23 ± 1.12^a^
FE	150.50 ± 5.20	0.47 ± 0.02	8.07 ± 0.21	58.79 ± 0.93^a^
FOL	148.50 ± 2.12	0.47 ± 0.01	7.91 ± 0.22	58.22 ± 1.30^a^
FEFOL	147.00 ± 4.24	0.46 ± 0.02	7.75 ± 0.25	59.06 ± 1.96^a^
	21 days			
C	147.33 ± 3.7^b^	0.48 ± 0.01^b^	7.98 ± 0.28	60.41 ± 1.69^b^
D	131.00 ± 6.68^a^	0.43 ± 0.01^a^	8.00 ± 0.43	56.58 ± 4.31^a^
FE	143.50 ± 5.00^ab^	0.44 ± 0.02^ab^	7.90 ± 0.16	57.19 ± 1.39^ab^
FOL	138.00 ± 9.90^ab^	0.43 ± 0.03^a^	7.73 ± 0.21	56.35 ± 2.28^a^
FEFOL	150.75 ± 4.57^b^	0.47 ± 0.02^ab^	8.04 ± 0.35	57.46 ± 2.11^ab^

*HB* hemoglobin, *HCT* hematocrit, *RBC* red blood cells, *MCV* mean corpuscular volume

^a,b,c^Significant differences between groups; ANOVA and Tukey’s test

**Table 6:** Concentration of iron (μg/g d.m.) in tissues in rats (mean and standard deviation)

Group	Liver	Spleen	Pancreas	Kidney	Heart
	2 days				
C	849.57 ± 186.62^b^	2332.88 ± 436.27^c^	63.03 ± 8.10^b^	250.84 ± 37.14^b^	291.61 ± 38.47^b^
D	171.38 ± 21.53^a^	716.51 ± 138.39^ab^	54.82 ± 13.41^ab^	155.02 ± 52.07^a^	267.06 ± 44.58^ab^
FE	788.47 ± 79.86^b^	1061.37 ± 362.10^b^	96.15 ± 16.72^c^	182.58 ± 22.99^a^	261.89 ± 28.33^ab^
FOL	187.69±62.30^a^	701.69 ± 126.22^ab^	49.79 ± 17.87^ab^	163.34 ± 12.05^a^	227.47 ± 42.86^a^
FEFOL	711.32 ± 164.23^b^	459.56 ± 91.72^a^	38.83 ± 14.26^a^	194.44 ± 28.54^a^	280.41 ± 76.31^ab^
	10 days				
C	853.68 ± 136.73^b^	2275.55 ± 573.84^c^	82.75 ± 24.68^b^	243.38 ± 33.86^b^	347.92 ± 78.64^b^
D	144.81 ± 21.89^a^	779.03 ± 213.42^a^	53.14 ± 6.26^a^	133.83 ± 17.52^a^	275.40 ± 55.36^a^
FE	1948.84 ± 343.78^c^	2176.46 ± 253.42^c^	61.48 ± 15.44^ab^	220.81 ± 27.80^b^	270.65 ± 30.00^a^
FOL	146.04 ± 43.38^a^	564.54 ± 83.76^a^	42.77 ± 12.56^a^	156.19 ± 16.29^a^	277.75 ± 33.22^a^
FEFOL	1100.29 ± 369.30^b^	1546.73 ± 218.26^b^	50.19 ± 21.74^a^	233.65 ± 23.94^b^	280.96 ± 13.47^a^
	21 days				
C	981.49 ± 256.92^b^	2759.40 ± 491.76^b^	89.00 ± 18.61^c^	263.95 ± 58.34^b^	305.85 ± 41.51
D	165.66 ± 34.26^a^	715.93 ± 121.25^a^	64.78 ± 12.30^b^	148.92 ± 13.34^a^	262.28 ± 27.89
FE	2417.41 ± 680.73^d^	3217.85 ± 307.84^c^	67.04 ± 14.82^b^	245.24 ± 21.14^b^	303.28 ± 34.41
FOL	150.64 ± 28.31^a^	673.94 ± 130.65^a^	39.64 ± 7.69^a^	146.94 ± 12.54^a^	284.74 ± 54.55
FEFOL	1796.61 ± 389.38^c^	2386.43 ± 375.42^b^	54.11 ± 12.49^b^	276.58 ± 16.25^b^	294.11 ± 24.87

^a,b,c^Significant differences between groups; ANOVA and Tukey’s test

We also compared the groups that differed by the amount of folic acid in the diet—that is, D with FOL and FE with FEFOL. We found that level of iron in the FEFOL group was significantly lower after 2 days in the pancreas, and after 10 and 21 days in the liver and spleen than in the FE group (Fig. 1). We also observed that the concentration in the FOL group was markedly lower in the spleen after 10 days and in pancreas after 10 and 21 days than in group D (Fig. 2).

## Discussion

In this study, we found that folate added to the diet affects tissue iron concentration in rats deficient in these micronutrients under conditions of both moderate and long-term supplementation. To the best of our knowledge, this is the first study to present such results.

The other important funding of this study is that iron concentration in the liver in deficient rats leveled out after only 2 days of supplementation with iron alone; in the spleen, pancreas, and kidneys, leveling took somewhat longer, lasting 10 days. Supplementation of both iron and folic acid extended the time for iron to replenish in the liver and spleen. Moreover, we observed that folate in the diet deficient in iron decreased iron concentration in the spleen and pancreas.

Iron deficiency causes anemia by production of smaller, less hemoglobinized erythrocytes [7], which was partly confirmed in this study, especially in groups D and FOL after 21 days of intervention. It seems that folic acid in deficient diet may improve erythropoietic activity and enhance iron utilization which may lead to iron redistribution from the liver and spleen to the erythropoietic process. However, in this study, we did not observed significant change in RBC between groups after supplementation in any stages. Hemoglobin concentration increased significantly only on 21 days in group FEFOL received iron with folic acid and this result with comparable amounts of RBC could indicate an increase in reticulocytes. Unfortunately, in this study we did not measured reticulocytes. Moreover, iron- and folate-deficient diet resulted in a decrease of MCV and only long-term supplementation improved MCV in FE and FEFOL groups. Administration of folic acid alone did not influence on MCV. Analysis of morphological parameters shows that folic acid may affect iron distribution and its utilization in rats; however, it appears that the obtained results also point to other mechanisms of folic acid effect on iron status.

Several studies have indicated that simultaneous supplementation of folic acid and iron does not improve iron status [13–15]. Srivastava et al. [13] found that 3-month supplementation with folic acid and iron did not significantly alter hemoglobin or ferritin concentration in women in the second trimester of pregnancy. Ahamed et al. [14] observed a slightly increase in hemoglobin and ferritin levels in pregnant women after 100 days of iron and folic acid supplementation, and their intervention did not reduce anemia in the study population. Serdula et al. [15] and Belay et al. [16] also observed a poor hemoglobin response in pregnant women after supplementation with iron and folic acid. The low level of compliance with folate and iron supplementation observed in some studies may be associated with folate and iron interactions. In this study, differences in iron contents between groups were seen only in tissues, and not in serum (the results were comparable). This can be explained by the maintenance of iron homeostasis in the blood while changes occurred within the distribution and storage of this microelement.

An important role in the regulation of iron homeostasis is played by the liver, especially by its regulation of hepcidin levels [17]. It is worth noting that a significant influence of folate on iron concentration was observed after 10 days of supplementation in the liver, spleen, and pancreas. Our results indicate that folic acid may decrease the bioavailability and distribution of iron in the organism. Although we did not examine all these mechanisms in our study, we found that during supplementation no interaction between iron and folic acid occurs on the level of duodenal transporters (unpublished data). It is certain that the mechanism that relates folate to iron status requires further investigation. Recent data have shown that multiple micronutrient supplementation (MMS) containing iron and folic acid is superior to Fe and folic acid supplementation alone. MMS in pregnancy reduced the risk of low birth weight, preterm birth, and being born small for gestational age; it also reduced the risk of neonatal and infant mortality [18].

Both efficiency and safety are important in iron supplementation [1, 19, 20]. On the basis of our results, we can conclude that folate decreases the efficiency of iron supplementation. However, the iron–folate combination may be beneficial for safety reasons, especially with long-term supplementation. We observed a great increase in iron content in the liver after 10 and 21 days of iron supplementation alone. Folate in the diet inhibited iron overload in the liver. Increased Fe accumulation in soft tissues has been demonstrated to be detrimental to their function [21]. Excess hepatic Fe storage has an effect on liver fibrosis, cirrhosis, liver dysfunction, and (in some cases) cancer [22]. It has been shown that Fe-dependent liver damage is associated with Fe-catalyzed oxidative stress [23]. Previous studies have found that oral iron supplementation improved iron status, but also increased oxidative stress, even in women with low iron stores [24, 25]. Interestingly, Tiwari et al. [26] observed that although treatment with iron and folic acid has remarkable efficacy in terms of hemoglobin, it may also lead to impaired liver function in pregnant anemic women. It has recently been shown that folic acid ameliorates liver inflammatory processes [27] and hepatic steatosis [28]. It seems that liver damage may by exacerbated by low serum folic acid concentrations, although the mechanism of this process is unknown [28]. This beneficial effect of folic acid is associated with its ability to regulate the transcription of genes related to hepatic oxidative stress [29] and 5′ AMP-activated protein kinase (AMPK) activity [30]. Folic acid may also have an impact on iron transporters in the liver, such as divalent metal-ion transporter-1 (DMT1) and ZRT/IRT-like protein 14 [31]. Hyeyoung et al. noted in Fe-loaded rats that hepatic ZRT/IRT-like protein 14 is twice as abundant as in Fe-adequate animals. The same study also determined that DMT1 levels in livers are lower in Fe-overloaded rats and higher in Fe-deficient animals [31].

This study has some limitations: First of all, we examined only one dose of iron and folate in the supplements and only one form of iron (iron gluconate). These data did not include other folic acid or iron status (such as ferritin and hepcidin) parameters. We did not assess the mechanism of interaction between iron and folic acid.

## Conclusion

Folic acid affects iron status in female rats deficient in these micronutrients in moderate and long-term supplementation. Thus, the use of iron supplements in combination with folates should be considered in terms of its effectiveness and safety.

## References

[1] Low MSY, Speedy J, Styles CE, de-Regil LM, Pasricha SR, Cochrane Developmental, Psychosocial and Learning Problems Group (2016). Daily iron supplementation for improving anaemia, iron status and health in menstruating women. Cochrane Database Syst Rev.

[2] Parisi F, Laoreti A, Cetin I (2014). Multiple Micronutrient Needs in Pregnancy in Industrialized Countries. Ann Nutr Metab.

[3] (2012) WHO | Guideline daily iron and folic acid supplementation in pregnant women. 23586119

[4] (2019) WHO | Intermittent iron and folic acid supplementation in adult women and adolescent girls. WHO

[5] Silva B, Faustino P (2015). An overview of molecular basis of iron metabolism regulation and the associated pathologies. Biochim Biophys Acta Mol basis Dis.

[6] Percy L, Mansour D, Fraser I (2017). Iron deficiency and iron deficiency anaemia in women. Best Pract Res Clin Obstet Gynaecol.

[7] Koury MJ, Ponka P (2004). New Insights Into Erythropoiesis: The Roles of Folate, Vitamin B _12_ , and Iron. Annu Rev Nutr.

[8] Laanpere M, Altmäe S, Stavreus-Evers A, Nilsson TK, Yngve A, Salumets A (2010). Folate-mediated one-carbon metabolism and its effect on female fertility and pregnancy viability. Nutr Rev.

[9] Van Der Woude DAA, De Vries J, Van Wijk EM (2014). A randomized controlled trial examining the addition of folic acid to iron supplementation in the treatment of postpartum anemia. Int J Gynecol Obstet.

[10] Hurrell R, Egli I (2010). Iron bioavailability and dietary reference values. Am J Clin Nutr.

[11] Skrypnik K, Suliburska J (2018). Association between the gut microbiota and mineral metabolism. J Sci Food Agric.

[12] Reeves PG (1997). Components of the AIN-93 diets as improvements in the AIN-76A diet. J Nutr.

[13] Srivastava R, Kant S, Singh A, Saxena R, Yadav K, Pandav CS (2019). Effect of iron and folic acid tablet versus capsule formulation on treatment compliance and iron status among pregnant women: a randomized controlled trial. J Fam Med Prim Care.

[14] Ahamed F, Yadav K, Kant S, Saxena R, Bairwa M, Pandav CS (2018). Effect of directly observed oral iron supplementation during pregnancy on iron status in a rural population in Haryana: A randomized controlled trial. Indian J Public Health.

[15] Serdula MK, Zhou Y, Li H, Liu JM, Mei Z (2018) Prenatal iron containing supplements provided to Chinese women with no or mild anemia had no effect on hemoglobin concentration in post-partum women or their infants at 6 and 12 months of age. Eur J Clin Nutr. 10.1038/s41430-018-0365-x 10.1038/s41430-018-0365-x 30446762

[16] Belay E, Endrias A, Alem B, Endris K (2018). Hematological responses to iron-folate supplementation and its determinants in pregnant women attending antenatal cares in Mekelle City Ethiopia. PLoS One.

[17] Rishi G, Subramaniam VN (2017). The liver in regulation of iron homeostasis. Am J Physiol Liver Physiol.

[18] Volani C, Paglia G, Smarason S, Pramstaller P, Demetz E, Pfeifhofer-Obermair C, Weiss G (2018). Metabolic signature of dietary iron overload in a mouse model. Cells.

[19] Koskenkorva-Frank TS, Weiss G, Koppenol WH, Burckhardt S (2013). The complex interplay of iron metabolism, reactive oxygen species, and reactive nitrogen species: Insights into the potential of various iron therapies to induce oxidative and nitrosative stress. Free Radic Biol Med.

[20] Brissot P, Ropert M, Le Lan C, Loréal O (2012). Non-transferrin bound iron: a key role in iron overload and iron toxicity. Biochim Biophys Acta, Gen Subj.

[21] Pietrangelo A (1996). Metals, oxidative stress, and hepatic fibrogenesis. Semin Liver Dis.

[22] Qiao Y, He H, Zhang Z, Liao Z, Yin D, Liu D, Yi B, He M (2016). Long-term sodium ferulate supplementation scavenges oxygen radicals and reverses liver damage induced by iron overloading. Molecules.

[23] King SM, Donangelo CM, Knutson MD, Walter PB, Ames BN, Viteri FE, King JC (2008). Daily supplementation with iron increases lipid peroxidation in young women with low iron stores. Exp Biol Med.

[24] Mani Tiwari AK, Mahdi AA, Chandyan S, Zahra F, Godbole MM, Jaiswar SP, Srivastava VK, Singh Negi MP (2011). Oral iron supplementation leads to oxidative imbalance in anemic women: A prospective study. Clin Nutr.

[25] Tiwari AKM, Mahdi AA, Mishra S (2018). Assessment of liver function in pregnant anemic women upon oral iron and folic acid supplementation. J Gynecol Obstet Hum Reprod.

[26] Sid V, Shang Y, Siow YL, Hewage SM, House JD, O K (2018). Folic acid supplementation attenuates chronic hepatic inflammation in high-fat diet fed mice. Lipids.

[27] Xia MF, Bian H, Zhu XP, Yan HM, Chang XX, Zhang LS, Lin HD, Hu XQ, Gao X (2018). Serum folic acid levels are associated with the presence and severity of liver steatosis in Chinese adults. Clin Nutr.

[28] Sarna LK, Wu N, Wang P, Hwang SY, Siow YL, O K (2012). Folic acid supplementation attenuates high fat diet induced hepatic oxidative stress via regulation of NADPH oxidase. Can J Physiol Pharmacol.

[29] Sid V, Wu N, Sarna LK, Siow YL, House JD, O K (2015). Folic acid supplementation during high-fat diet feeding restores AMPK activation via an AMP-LKB1-dependent mechanism. Am J Physiol Integr Comp Physiol.

[30] Nam H, Wang CY, Zhang L, Zhang W, Hojyo S, Fukada T, Knutson MD (2013). ZIP14 and DMT1 in the liver, pancreas, and heart are differentially regulated by iron deficiency and overload: Implications for tissue ironuptake in iron-related disorders. Haematologica.

[31] Sudfeld CR, Smith ER (2019). New evidence should inform WHO guidelines on multiple micronutrient supplementation in pregnancy. J Nutr.

